# Statin therapy, myopathy and exercise--a case report

**DOI:** 10.1186/1476-511X-11-40

**Published:** 2012-03-16

**Authors:** Stuart J Semple

**Affiliations:** 1 Department of Biokinetics & Sport Science, University of Zululand, KwaDlangezwa, South Africa

**Keywords:** Lipids, Inflammation, Exercise, Simvastatin

## Abstract

In a bid to reduce the morbidity and mortality associated with coronary artery disease, statin therapy has become a cornerstone treatment for patients with dyslipideamia. Statins, or HMG-CoA reductase inhibitors, are effective in blocking hepatic synthesis of cholesterol and are generally regarded as safe. Although rare, severe adverse side effects such as rhabdomyolysis have been reported, however, the more common complaint from patients is that related to myopathy. There is also mounting evidence that exercise may exacerbate these side effects, however the mechanisms are yet to be fully defined and there is controversy regarding the role that inflammation may play in the myopathy. This paper reports a patients experience during 6 months of simvastatin therapy and provides some insight into the white cell count (inflammation) following two bouts of moderate intensity exercise before and during statin therapy. It also highlights the need for rehabilitation practitioners to be aware of the adverse effects of statins in exercising patients.

## Introduction

Reducing high cholesterol levels through pharmacotherapy is a key goal for patients with dyslipedemia. Despite the controversy surrounding statins they remain one of the most widely prescribed groups of lipid-lowering drugs simply because of their effectiveness [1]. Serious adverse side effects are rare, however, myopathy symptoms including fatigue, weakness, cramps and muscle pain are commonly reported by patients [2]. These symptoms may be exacerbated in patients who exercise [2] and be prevalent in as many as 75% of athletes who take the drug [3]. The underlying mechanism(s) responsible for the statin-induced myopathy is unclear, however there is evidence that statins may upregulate muscle cell apoptosis, inflammation and protein catabolism in response to eccentric exercise [4]. Whether or not 6 months of statin therapy induces skeletal muscle related changes such that indirect markers of muscle damage are exacerbated following 'normal' sessions that induce moderate muscle soreness, is yet to be established. Indeed some will argue that there are currently very few studies implicating inflammation as a factor that may exacerbate statin therapy induced complications [5].

## Patient background

**Figure 1:**
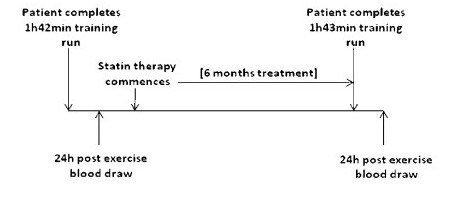
**Case Study Design**.

A 34 year old male (weight 63 kg; BMI 21.8 kg/m^2^) presented to his general practitioner (GP) for a routine medical check-up. The patient, a non-smoker, had no known/diagnosed chronic disease, injury or infection, was on no medication and was generally in good health. As a junior athlete the patient had participated competitively in endurance activities (cross-country, athletics, tri/duathlon) at provincial and national level, and for the last 13 years his motivation to engage in regular physical activity was driven by the health belief model. On average, his training over the last few years included 3 road running sessions per week (2 × 30-40 min during the week and 1 × 60-100 min on the weekend). These sessions were usually completed at moderate intensity with the odd session (1 out of every 4) being conducted at high intensity. He has a family history of cardiovascular disease (hypertension and dyslipidemia). 24 h prior to the GP consult he had completed a training run of 1 h 42 min (Figure 1). On receiving the pathology results from the routine medical examination he was prescribed 10 mg simvastatin and advised to reassess the lipoprotein levels after 6 months of treatment. Ethics approval for this case study was granted from the institutions Faculty of Science & Agriculture Ethics Committee and the research complied with the Declaration of Helsinki.

Three weeks after initiating the treatment the patient participated in a 21.1 km road race. He exerted himself harder than usual on an undulating course (finishing time 1 h 40 min). It was following this event that the he started to report muscle aches during rest. The event seemed to 'trigger' the symptoms of myopathy and the patient also reported that it took an "unusually long time to recover". The patient subsequently reported heavy legs during all training sessions regardless of the intensity and continued to experience myalgia for approximately 3 months. The intermittent discomfort at rest, together with the sensation of heavy legs whilst training, was pronounced enough for the patient to query these symptoms with his GP who recommended changing from simvastatin to a different statin. For the purposes of this case study the patient remained on the simvastatin. Importantly the patients pre-statin exercise regimen remained the same as for the duration of the 6 months before the second blood draw. Once again he completed the same route and distance in a comparable time (1 h 43 min 40 s) 24 h prior to the second blood draw. The pre-exercise meal and fluid ingestion during both runs was the same.

## Discussion

The effectiveness of the statin therapy in reducing cholesterol levels in this patient is evident in Table 1. The patient did not make any modifications to his diet during the 6 months. Although still not within the target range, total cholesterol decreased by 14%, triglycerides and LDL's decreased by 18% and 14% respectively. White cell count was elevated after run 2 by 8%, with a 75% elevation in lymphocytes and a 1200% elevation in eosinophils. More modest elevations were observed for neutrophils following the second run (1.6%). The increased white cell count following the statin therapy may suggest that inflammation was up-regulated following run 2. White cell counts are commonly used to identify or measure exercise-induced inflammatory responses [6]. Recently Boutibir et al. [7] showed that statin therapy in rats significantly reduces exercise capacity and that the more pronounced reactive oxygen species (ROS) production that they observed during the exercise may be responsible for the finding. Similarly, another recent study by Kwak et al. [8] has shown that simvastatin increases oxidative stress and atrophy in human muscle cells. Based on these findings and those of the current case study it would seem reasonable to suggest that statin therapy may induce changes in human muscle such that ROS production during exercise increases with a concomitant increase in inflammation. Statins exhibit potent anti-inflammatory properties [9]. What then may be the reason for the observed increase in WBC levels following exercise in this patient? Creatine kinase, another indirect marker of muscle damage is significantly elevated in statin users following exercise when compared to non-statin users [10]. Urso et al. [11] have hypothesized that statin therapy may negatively affect the stability of skeletal muscle cell membranes. This may in part explain why CK is elevated following exercise, particularly strenuous or unaccustomed exercise. The more pronounced muscle damage may up-regulate inflammation which in turn could account for the longer recovery time that exercising patients experience.

**Table 1:** Pre and 6 month post statin therapy lipoprotein and white blood cell concentrations

	Pre-statin therapy^♦^	6 months post-statin therapy^♦^	Normative range
Cholesterol (total)	7.1	6.1	2.8 - 4.9 mmol/l

Triglycerides	2.2	1.8	0.5 - 1.6 mmol/l

HDL Cholesterol	1.5	1.5	1.0 - 1.6 mmol/l

LDL Cholesterol	4.9	4.2	1.6 - 2.9 mmol/l

White cell count	5.65	6.10	3.92 - 9.88 10^^^9/l

Neutrophils	3.03	3.08	2.00 - 7.50 10^^^9/l

Lymphocytes	1.32	2.32	1.00 - 4.00 10^^^9/l

Monocytes	0.37	0.37	0.18 - 0.80 10^^^9/l

Basophils	0.09	0.05	0.00 - 0.20 10^^^9/l

Eosinophils	0.02	0.27	0.00 - 0.45 10^^^9/l

ESR	2	2	2 - 2.8 mm/hr

Creatinine	93	95	64 - 104 umol/l

^♦ ^Fasting samples obtained 24 h after a 20 km training run; HDL = high density lipoproteins; LDL = low density lipoproteins; ESR = erythrocyte sedimentation rate

The patient did report the occurrence and severity of aching muscles and heavy legs to subside after 3 months but these symptoms did not resolve completely. In addition the patient reported that the myopathy during, and recovery time from flu was gauged to be worse and longer than normal. Despite myopathies being the most commonly reported side effect of statin therapy the patient was not informed of the potential side effects of the drug until he consulted his GP after 3 months of therapy. Clinicians and rehabilitation practitioners should be aware of the prevalence and potential negative effects that statin therapy has on patients (even in low doses) and in particular on those patients that regularly engage in physical activity. It is arguable that this is even more pertinent for patients who were previously sedentary and then begin exercising as part of their chronic disease rehabilitation plan. The paradox which exists for these patients is that physical activity which is supposed to improve functionality and quality of life may actually exacerbate the myopathy if they are taking statins. This makes adherence to physical rehabilitation programmes a challenge. There are also practical implications for programme design by rehabilitation practitioners. Longer rest periods between training sessions and/or alternating between activities that stress different musculature and metabolic pathways may be required especially during the first 10-12 weeks of exercising. To date there is very little information available on inflammatory responses to exercise before and following long term treatment with statins. The measurement of leukocyte free radical generation or leukocyte function prior to and after statin therapy with and without exercise exposure would provide greater insight into the association between inflammation, exercise and myopathy. Unfortunately, for this case study, the patients routine pathology tests did not include such measures, however the results do tend to suggest that quantitatively there is a change in the inflammatory response, and in the absence of alterations to diet, physical activity patterns or other known pathology it would seem reasonable to suggest that the statin treatment is linked to this observation.

## Abbreviations

GP: General practitioner; HDL: High density lipoproteins; LDL: Low density lipoproteins; ESR: Erythrocyte sedimentation rate; ROS: Reactive oxygen species; CK: Creatine kinase.

## Competing interests

The author declares that they have no competing interests.
